# Modulating Antiangiogenic Resistance by Inhibiting the Signal Transducer and Activator of Transcription 3 Pathway in Glioblastoma

**DOI:** 10.18632/oncotarget.663

**Published:** 2012-09-19

**Authors:** John de Groot, Ji Liang, Ling-Yuan Kong, Jun Wei, Yuji Piao, Gregory Fuller, Wei Qiao, Amy B. Heimberger

**Affiliations:** ^1^ Department of Neuro-Oncology, The University of Texas M.D. Anderson Cancer Center, Houston, TX; ^2^ Department of Neurosurgery, The University of Texas M.D. Anderson Cancer Center, Houston, TX; ^3^ Department of Neuropathology, The University of Texas M.D. Anderson Cancer Center, Houston, TX; ^4^ Department of Biostatistics, The University of Texas M.D. Anderson Cancer Center, Houston, TX

**Keywords:** bevacizumab, anti-vascular endothelial growth factor, signal transducer and activator of transcription 3, treatment failure, invasion

## Abstract

Determining the mechanism of treatment failure of VEGF signaling inhibitors for malignant glioma patients would provide insight into approaches to overcome therapeutic resistance. In this study, we demonstrate that human glioblastoma tumors failing bevacizumab have an increase in the mean percentage of p-STAT3-expressing cells compared to samples taken from patients failing non-antiangiogenic therapy containing regimens. Likewise, in murine xenograft models of glioblastoma, the mean percentage of p-STAT3-expressing cells in the gliomas resistant to antiangiogenic therapy was markedly elevated relative to controls. Administration of the JAK/STAT3 inhibitor AZD1480 alone and in combination with cediranib reduced the infiltration of VEGF inhibitor-induced p-STAT3 macrophages. Thus, the combination of AZD1480 with cediranib markedly reduced tumor volume, and microvascular density, indicating that up regulation of the STAT3 pathway can mediate resistance to antiangiogenic therapy and combinational approaches may delay or overcome resistance.

## INTRODUCTION

Patients with glioblastoma (GB) invariably develop recurrence or disease progression, limiting their median survival time to only 14.8 months despite multimodality therapy [1]. Bevacizumab, a humanized monoclonal antibody that sequesters vascular endothelial growth factor (VEGF), has been approved for treatment of recurrent GB based on the radiographic endpoint of the reduction in gadolinium-enhancement seen on T1-weighted magnetic resonance images, defining a prolongation of progression-free survival [2]. Although there can be a marked reduction in the contrast-enhancing component of the tumor, progression can be detected by either an increase in the abnormal fluid-attenuated inversion recovery (FLAIR) image hyperintensity, reflective of nonenhancing tumor infiltration [3, 4], or a re-establishment of contrast enhancement, reflecting VEGF-independent angiogenesis. VEGF signaling inhibitors have been shown to rapidly decrease vascular permeability which manifests as a decrease in contrast enhancement that does not correlate with tumor size [5]. Upon failure of bevacizumab, very few subsequent responses to salvage chemotherapy have been reported [3], suggesting the emergence of a highly treatment-resistant phenotype. VEGF signaling inhibitor approaches have been shown to increase the invasion of the brain parenchyma by glioma cells [6, 7]. Thus, it has been postulated that treatment with antiangiogenic therapy selects for the emergence of the infiltrative, VEGF-independent components of GBs, for which current therapies are not adequate.

Hypoxia is a predominant characteristic of GB [8], and other investigators have suggested that VEGF signaling inhibitor treatment can reduce the blood supply within GB, leading to a more hypoxic environment [9]. Hypoxia has been shown to promote the attraction of bone marrow derived myeloid cells to hypoxic glioblastoma tumors [10]. Moreover, we have recently demonstrated that hypoxia can induce the expression of p-STAT3 in glioma cells [11]. STAT3 activation usually relies on ligand-receptor interactions [12], but STAT3 becomes persistently activated in most human malignancies, including gliomas [13]. STAT3 activation entails protein phosphorylation at the tyrosine-705 site (p-STAT3), dimerization, nuclear translocation, and subsequent binding to consensus promoter sequences of target genes, thus initiating transcription of multiple genes fundamental to tumor progression including preventing apoptosis, and enhancing proliferation, angiogenesis, and invasion [14]. Additionally, STAT3 maintains the “stemness” of glioma cells [15], the mesenchymal transformation of brain tumors [16] and tumor-mediated immune suppression [17]. Finally, STAT3 has been shown to be a key defining feature responsible for the polarization of the tumor-associated macrophage to the tumor supportive M2-phenotype [18]. *We therefore hypothesized that antiangiogenic therapy would induce the expression of STAT3 expression in the glioma tumor and its microenvironment during progression and inhibiting its expression would enhance the efficacy of anti-VEGF therapy*.

## RESULTS

### Up Regulation of p-STAT3 in GB Patients During Treatment Failure of Bevacizumab

**Figure 1:**
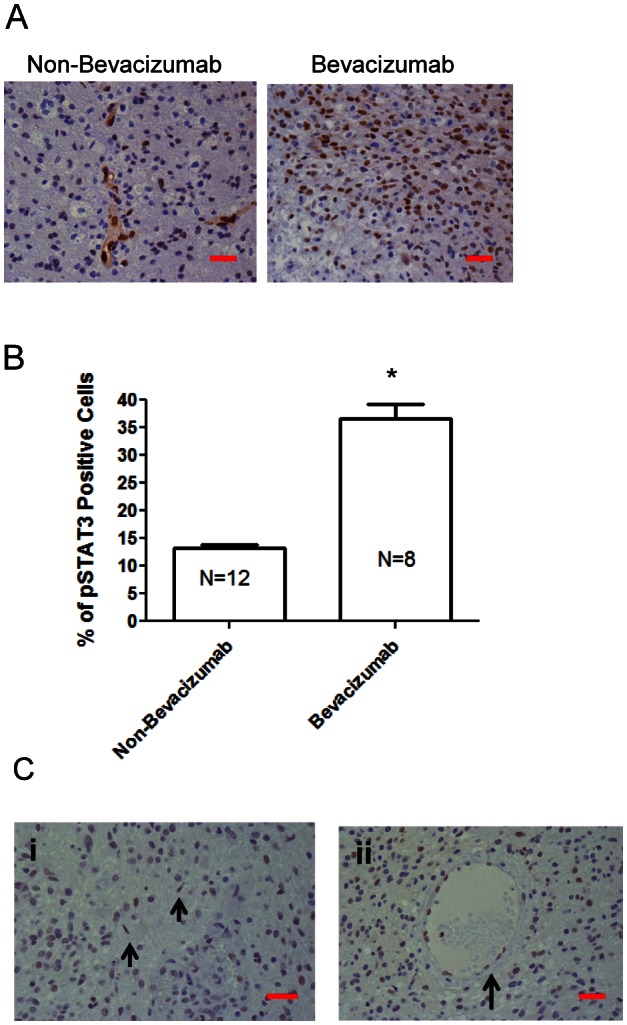
The p-STAT3 pathway is up regulated during treatment failure of anti-VEGF therapies in human GB patients (A) Representative light microscopy images showing immunohistochemical detection of p-STAT3 (brown nuclear staining) in a patient with recurrent GB who was treated with temozolomide compared with a similar patient treated with bevacizumab (400x). Although there is heterogeneous p-STAT3 expression within any given glioma, images shown are typical representations of the entire tumor. (B) Bar graph demonstrating the average percentage of p-STAT3-positive glioma cells in patients with recurrent GB who were treated with various alternative chemotherapeutics compared with similar patients treated with bevacizumab. *P=0.0008. (C) Representative light microscopy images showing immunohistochemical detection of p-STAT3 in glioma from a patient treated with bevacizumab demonstrating positive p-STAT3 nuclear expression in microglia cells (denoted by arrows in panel i) and endothelial cells (denoted by arrow in panel ii) (all at 400x). The red size bars are 50 microns.

To ascertain if p-STAT3 expression was increased during treatment failure with bevacizumab, a retrospectively, randomly identified group of patients with recurrent GB for whom failed either conventional chemotherapy (n=12) or treatment with bevacizumab (n=8) and who had undergone subsequent resection or biopsy, was analyzed for expression of p-STAT3 (Fig. 1A; Supplementary Table 1). For the bevacizumab-treated patients, the tumors had been resected 30-60 days post administration of bevacizumab as recommended by the package insert. Patients with recurrent GB who were treated with bevacizumab had a mean of 36.7 + 7.4% p-STAT3 expressing cells in comparison to a recurrent glioblastoma cohort that had never received any type of VEGF signaling inhibitor therapy that had a mean of 13.3 + 1.6% p-STAT3 expressing cells (P=0.0008 by student t-test) (Fig. 1B). Furthermore, based on available specimens, there was a trend in the increase of glioma p-STAT3 expression to a mean of 35.5 + 10.3% at recurrence in the bevacizumab-treated patients relative to the original, pre-treatment matched specimens that had a mean of 14.8 + 7.6% p-STAT3 expressing cells (P= 0.08 by student t-test) (Supplementary Figure 1). Neuropathological review indicated that the p-STAT3 nuclear expression was not only seen in the pleomorphic tumor cells, but also within cells with thin, elongate, nuclei with slender bipolar cytoplasmic processes that have the morphological characteristics of microglia, and in the endothelium (Fig. 1C). The nature of p-STAT3 expression in the diffusely infiltrating component can't be evaluated since this is not routinely resected.

### Up Regulation of p-STAT3 in Treatment Failure in Orthotopic Model Systems of Glioma

**Figure 2:**
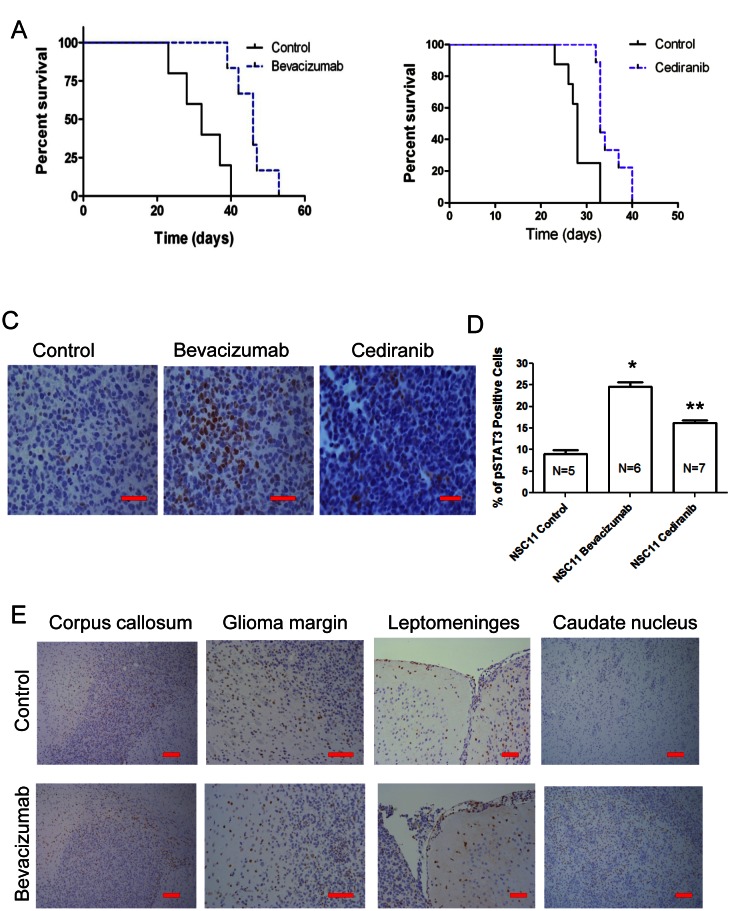
VEGF signaling inhibitors enhance survival in murine model systems but upon treatment failure the p-STAT3 pathway is up regulated (A) Graph showing Kaplan-Meier estimate of survival showing improved survival in nude mice with NSC11 tumors treated with bevacizumab compared with untreated controls. *P*<0.0025. (B) Graph showing Kaplan-Meier estimate of survival showing improved survival in nude mice with NSC11 tumors treated with cediranib compared with untreated controls. P=0.0021. (C) Representative light microscopy images showing immunohistochemical detection of p-STAT3 (brown nuclear staining) in NSC11 tumors from a control mouse (treated with vehicle alone), a bevacizumab-treated mouse, and a cediranib-treated mouse (400x). (D) Bar graph demonstrating the average percentage of p-STAT3-positive glioma cells in a orthotopic model of intracerebral glioma (NSC11) treated either with vehicle (control), bevacizumab, or cediranib. *P=0.0011, **P=0.0125. (E) Representative light microscopy image showing immunohistochemical detection of p-STAT3 (brown nuclear staining) in the contralateral corpus callosum within a control- and bevacizumab-treated mouse (100x), at the glioma margin (200x), along the contralateral leptomeninges (200x), and in the contralateral caudate nucleus interspersed among white matter tracts (100x).

To determine if up-regulation of p-STAT3 expression is recapitulated in orthotopic models of glioma at the time of antiangiogenic treatment failure, expression of p-STAT3 nuclear expression was evaluated. Mice treated with bevacizumab or cediranib were evaluated to ascertain whether findings represent a more generalized mechanism of escape to anti-angiogenic therapy. We treated mice with intracerebral tumors with anti-VEGF therapy starting on day 5 after tumor cell implantation. The median survival time for the NSC11 tumor-bearing mice treated with the control was 32 days. In contrast, the NSC11 tumor-bearing mice treated with bevacizumab had a median survival time of 46 days, which was statistically significant (*P*<0.0025; HR= 16.55) (Fig. 2A). To ascertain whether any potential findings were specific to bevacizumab or were a more generalized propensity of VEGF signaling inhibitors, the pan-VEGFR targeting agent, cediranib, was also evaluated. In the NSC11 tumor-bearing mice treated with cediranib, the median survival time was 33 days, which was significantly greater than that of the control cohort (28 days; P=0.0021; HR = 9.38) (Fig. 2B).

The tumors during treatment failure from either the control group or the bevacizumab- or the cediranib-treated group were then compared (Fig. 2C). In the control-treated NSC11 gliomas, the mean percentage of p-STAT3 expressing tumor cells was 9.0 + 4.4% (median 6.4; range: 5.1 – 14.0; n=5), whereas in the bevacizumab-treated cohort, the mean was 24.6 + 6.4% (median 22.7; range: 16.0 – 33.3; n=6; *P*=0.008 by pair comparison). Cediranib-treated NSC11 glioma cells also showed a significant elevation of p-STAT3 expression, with a mean percentage of p-STAT3-expressing cells of 16.2 + 4.8% (median 16.5; range 10.0 – 22.4; n=7; *P*=0.05) (Fig. 2D). The overall comparison among the three groups of treated NSC11 tumors indicated there is significant difference (P=0.004 by Kruskal Wallis test).

In addition to the quantitative difference of p-STAT3 tumor expression between the control and the antiangiogenic therapy-treated group, there was a distinctive difference in the pattern of p-STAT3 expression. In the control (n=5) and bevacizumab-treated (n=6) groups at the time of treatment failure, STAT3-expressing cells could be seen at the leading edge of the corpus callosum, at the tumor margin and along the leptomeninges. However, there were more p-STAT3-positive cells diffusely infiltrating into the contralateral caudate nucleus in the bevacizumab treatment group (Fig. 2E) suggesting p-STAT3 may be an important signaling mediator in these highly invasive tumors.

### Cediranib and AZD1480 Reduce Glioma Tumor Volume

The median survival time for the NSC11 tumor-bearing mice treated with the combination of bevacizumab and AZD1480 was 42.5 days, which compared favorably to the bevacizumab-treated group with a median survival time of 39.5 days, the AZD1480-treated group with a median survival time of 34 days and the control group with a median survival time of 28 days (*P*=0.016 by Log-rank comparison of survival curves)(Supplementary Figure 2).

**Figure 3:**
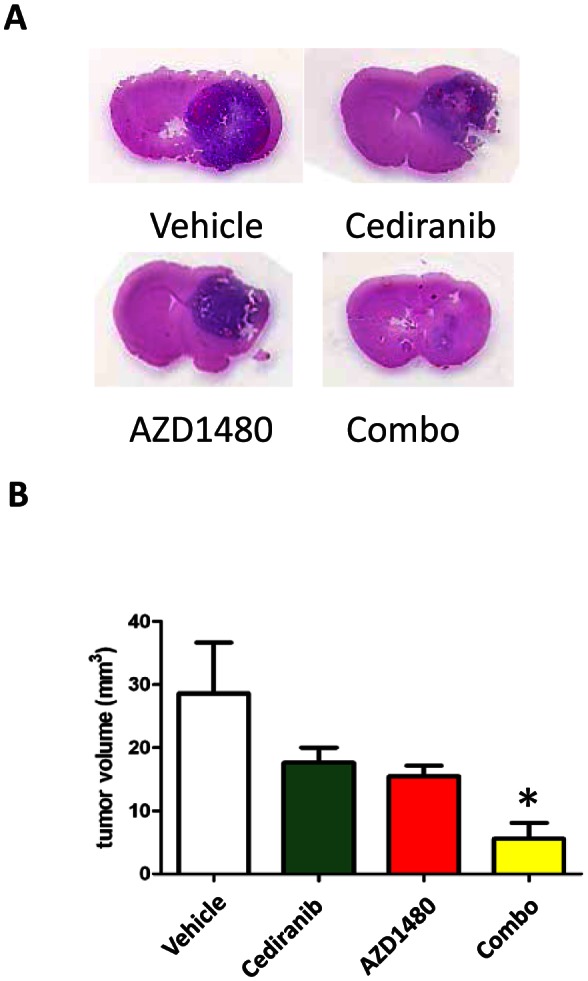
STAT-3 blockade with cediranib reduces glioma volume (A) Representative whole mounts of H & E stained brains containing GL261 tumors in C57BL/6J mice treated with vehicle control, cediranib, AZD1480 or the combination after 14 days of treatment). (B) Bar graph demonstrating the volume of intracranial GL261 tumors in C57BL/6J mice (n=7/group). *P= 0.02.

Since bevacizumab targets human VEGF, therapeutic effects against murine tumors would be anticipated to be marginal. As both a proof of principal evaluation in an immunocompetent animal model and the logistic feasibility of a combinational approach being implemented in human clinical trials, cediranib and AZD1480 (both owned by AstraZeneca) were tested in the immune competent syngeneic GL261 glioma model. Thus, C57BL/6J mice were injected with GL261 cells and then treated with each agent or the combination for 28 days (Fig. 3A). The mean tumor volume in the control group was 29 + 9 mm^3^, whereas it was 18 + 3 mm^3^ in the cediranib-treated and 15 + 2 mm^3^ in the AZD1480-treated group (*P* = 0.23 and 0.18, respectively compared with the control group). The combination of both cediranib and AZD1480 resulted in marked tumor volume suppression to a mean volume of 6 + 3 (*P* = 0.02; Fig. 3B).

### AZD1480 Reduces Glioma Tumor Vasculature and Hypoxia

**Figure 4:**
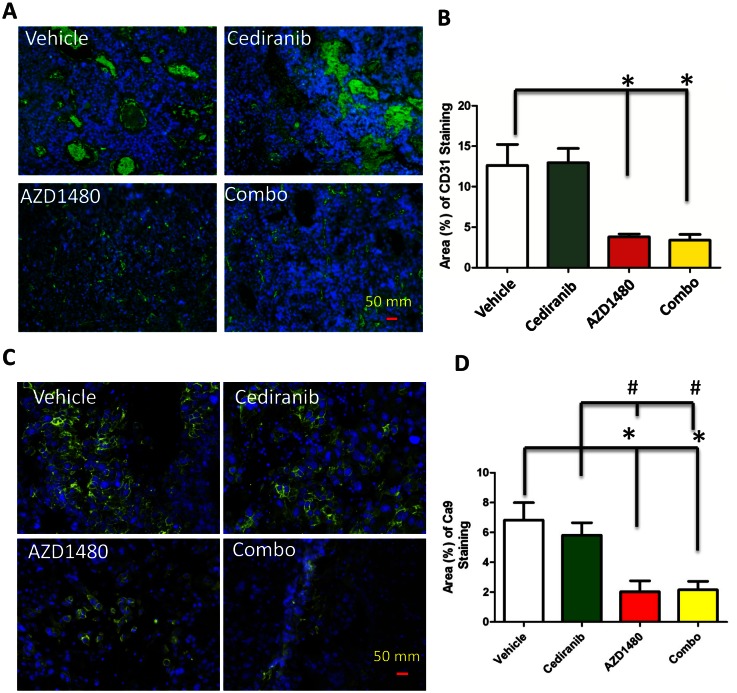
(A) Representative light microscopy images showing immunohistochemical detection of CD31 (green) in GL261 gliomas Cell nuclei were counterstained with DAPI (4', 6-diamidino-2-phenylindole) (blue). All images were taken at 400 x. (B) Bar graph demonstrating the mean vascular density in GL261-treated tumors. *P=0.015. (C) Representative light microscopy images showing the membrane expression of hypoxia marker carbonic anhydrase 9 (CA9; green) in the four treatment groups. Cell nuclei are counterstained with DAPI (4', 6-diamidino-2-phenylindole) (blue). (D) Quantitation of CA9 staining as the percentage of CA9 staining in each treatment group. * P<0.01 compared to control and # P<0.01 compared to cediranib-treated animals.

Since p-STAT3 expressing macrophages have been shown to induce angiogenesis [19], we next determined if AZD1480 could inhibit VEGF-independent angiogenesis induced by pan-VEGFR blockade by cediranib. After 14 days of treatment, the vascular size of tumor infiltrating vessels was modestly decreased in the cediranib-treated group but was markedly inhibited in the AZD1480-treated group (Fig. 4A). The AZD1480-induced diminution of vascular caliber was of such degree that further reduction was not detected in tumors treated with the combination of cediranib and AZD1480 (P =0.015; Fig. 4B). This indicates a switch from macro- to microvessels in the AZD1480 group.

Since antiangiogenic therapy-induced vascular pruning is suspected to modulate tumor hypoxia in glioblastoma and other solid tumors and STAT-3 is known to activate and stabilize HIF-1α [20] and promote VEGF expression, we evaluated tumor hypoxia in tumors at the time of control and cediranib treatment failure (Fig. 4C). Compared to control and cediranib-treated tumors, AZD1480 significantly reduced tumor hypoxia by 71 and 66%, respectively as assessed by CA9 staining (P<0.01, Fig. 4D). AZD1480 minimized hypoxia induction when used in combination with cediranib where there was a similar 69% and 64% reduction in CA9 staining compared to control and cediranib-treated tumors, respectively (P<0.01, Fig. 4D).

### AZD1480 Inhibits Cediranib Enhanced Macrophage Infiltration

**Figure 5:**
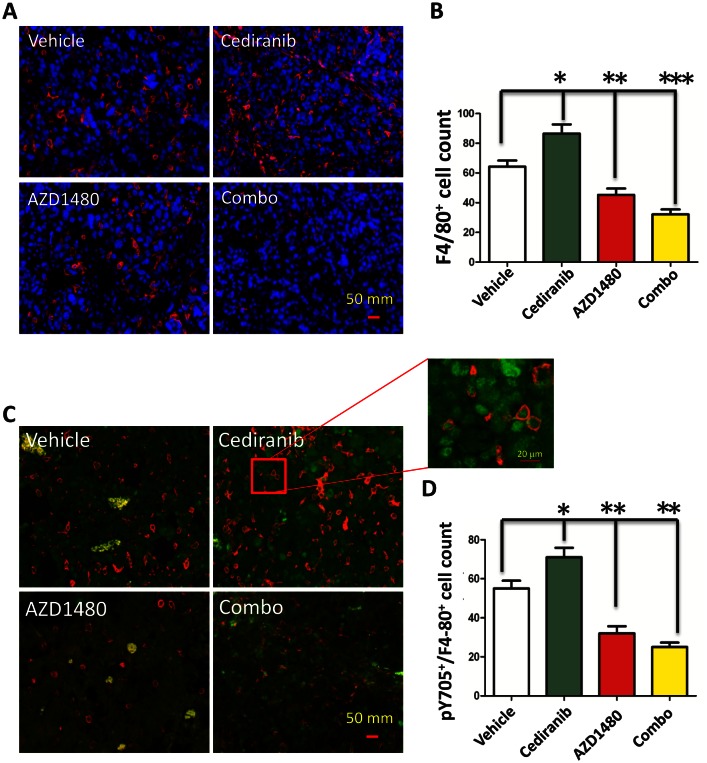
STAT-3 blockade reduces macrophage infiltration (A) Representative light microscopy images showing immunohistochemical detection of macrophages in GL261 gliomas as detected with an anti-F4/80 antibody (red). Cell nuclei were counterstained with DAPI. All images were taken at 400 x. (B) Bar graph demonstrating the average percentage of F4/80-positive cells in the GL261 murine model system treated either with vehicle (control), cediranib, AZD1490 or the combination. *P= 0.01, **P=0.007, ***P<0.0001. (C) Representative light microscopy images showing immunohistochemical detection of macrophages (red) and intranuclear p-STAT3 (green). All images were taken at 400 x. (D) Bar graph demonstrating the average percentage of F4/80-positive cells in the GL261 murine model system treated either with vehicle (control), cediranib, AZD1490 or the combination. *P=0.001, **P<0.0001

Previously treatment of human patients with bevacizumab has been shown to induce a marked increase in the influx of macrophages [21]. To ascertain if cediranib similarly induces enhanced macrophage infiltration that could be potentially blocked with AZD1480, the GL261 tumors were fluorescently labeled with the macrophage marker F4/80 (Fig. 5A). In the cediranib-treated tumor the mean number of F4/80^+^-infiltrating cells was 86.67 + 6.65 cells/high powered field (HPF) which was a 35% increase compared to vehicle control tumors that had a mean number of 64.29 + 4.43 (P=0.01; Fig. 5B). There was modest decrease in the AZD1480-treated group to a mean of 45.25 + 4.73 and this change was statistically significant (P=0.007). However, the combination of both cediranib and AZD1480 resulted in a marked reduction in the number of glioma-infiltrating macrophages to 32.13 + 2.13 (P<0.0001 compared to vehicle control). Further characterization of these macrophages demonstrated that they expressed p-STAT3 (Fig. 5C) indicating a M2 tumor-supportive phenotype and that cediranib and AZD1480 in the combination treated group was preferentially reducing this subpopulation (i.e. M2 immune suppressive macrophages) (Fig.5D). Specifically, in the cediranib-treated tumors the mean number of p-STAT3^+^F4/80^+^-infiltrating cells was 71 + 5.29 which was a 29% increase compared to vehicle control tumors that had a mean number of 55 + 4.34. In both the AZD1480-treated group and the combination of both cediranib and AZD1480 resulted in a marked reduction in the number of p-STAT3^+^F4/80^+^-infiltrating cells to 32.13 + 2.13 and 25 + 1.54, respectively (P=0.001 and P<0.0001, respectively compared to vehicle control).

### Reduction of nestin expression with the combination of cediranib and AZD1480

**Figure 6:**
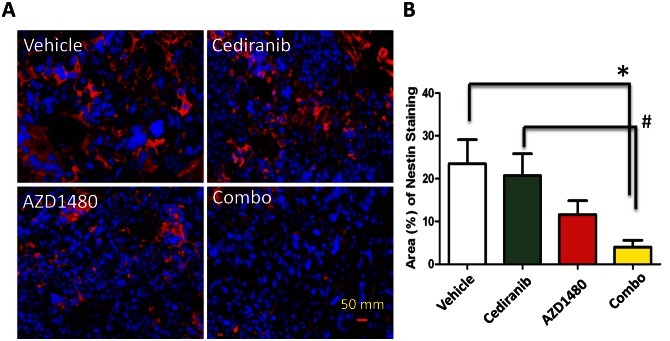
STAT-3 blockade in GL261 tumors treated with cediranib blocks the maintenance of stem cell properties (A) Representative light microscopy images showing immunohistochemical detection of nestin (red) and nuclei (blue). All images were taken at 400 x. (B) Bar graph demonstrating the average percentage of nestin-positive cells in the GL261 murine model system treated either with vehicle (control), cediranib, AZD1490 or the combination. *P=0.008, #P=0.009.

Finally, since p-STAT3 has been associated with the maintenance of stem cells, we assessed the expression of nestin in the various treatment groups in GL261 in the C57BL/6J background (Fig. 6A). The selection of nestin was based on the fact that it has been used extensively as a marker of CNS progenitors and stem cells within various areas of the developing nervous system [22-26] but is also expressed in newly formed blood vessels [27]. In the cediranib-treated tumors, the mean number of nestin-expressing cells was 20.70 + 5.53% which was similar to the vehicle control that had a mean number of 23.47 + 6.05% (P=0.73; Fig. 6B). There a trend in the reduction of nestin-expressing cells in the AZD1480-treated group to a mean of 11.62 + 3.50% (P=0.1 compared to the vehicle control) but there was a marked reduction in nestin staining to a mean of 4 + 1.76% in the combination treated group (P=0.008 compared to vehicle control and P=0.009 compared to the cediranib-treated group).

## DISCUSSION

In human glioblastoma patients and in orthotopic mouse models of glioblastoma, we have demonstrated that one potential mechanism of treatment failure of VEGF-targeted antiangiogenic agents is the up regulation of p-STAT3 that is associated with tumorigencity and enhanced progression that is consistent with the aggressive phenotype that has been previously described upon failure with anti-VEGF agents [3, 9, 28]. The lack of increased angiogenesis as reflected by CD31 count and tumor hypoxia as reflected by CA9 expression, in the setting of treatment failure of cediranib, was contrasted by a statistically significant increase in p-STAT3 expressing macrophages. This data would indicate a role for the p-STAT3 expressing, tumor-supportive macrophage (i.e. M2) [19] during treatment failure. AZD1480 decreased total tumor p-STAT3 cells and this was most evident in the macrophage population. When the STAT3 pathway is blocked in the setting of VEGF inhibition in mice with intracerebral gliomas, glioma volume is reduced and the additive effect correlated with inhibition of nestin and glioma-infiltrating macrophages. This would be consistent with the previously documented role of glioma-infiltrating myeloid cells such as macrophages and microglia playing a central role in the angiogenic and invasive mechanisms of resistance by the STAT3 pathway [29] and having a negative prognostic influence on survival [30]. This study illustrates that anti-angiogenic failure is not solely a property of the glioma cell but also of the tumor supportive microenvironment, specifically the tumor-associated macrophage that must also be therapeutically addressed. The mechanism of activity of the combination of cediranib and AZD1480 however is not exclusively confined to the inhibition of STAT3 expressing macrophages in the glioma since the combinational approach also inhibited nestin.

A role of myeloid derived cells (precursors of monocytes, macrophages and granulocytes) in mediating refractoriness to antiangiogenic therapy has been previously demonstrated [31]. Refractory tumors were associated with a significant increase of tumor infiltrating myeloid cells relative to the sensitive tumors and these myeloid cells were capable of promoting angiogenesis independent of VEGF. Thus, tumors can direct the bone marrow to increase myeloid cell production and enhance the recruitment of these cells to the tumors [32, 33]. Although these investigators did not address differences in hypoxia between these animal models, their data implicated multiple mechanisms are likely involved. Cumulatively, these findings are consistent with our findings of increased STAT3-expressing macrophages in the cediranab-treated cohort during treatment failure, albeit in a derivative immune population.

MR imaging of GBs 24 hours after bevacizumab administration have demonstrated a marked reduction in the total number of perfused vessels likely resulting in a rapid reduction of cerebral permeability and/or perfusion and a subsequent hypoxic state [34]. Antiangiogenic therapy-mediated induction of hypoxia within the tumor microenvironment could be directly responsible for activation of the STAT3 pathway and the chemoattraction of myeloid cells to the tumor via STAT3-mediated mechanisms. Tumor hypoxia, via HIF-2α, has been shown to increase the trafficking of macrophages into the tumor microenvironment and increase the tumor burden and the progression of malignancy [35] potentially through the hypoxia-induced CREB-C/EBPβ cascade [36]. Both hypoxia and antiangiogenic therapies such as cediranib have been shown to induce the attraction of myeloid cells to tumors, likely via multiple mechanisms. However, at the time of treatment failure, when this study analysis was conducted, we did not detect an increase in hypoxia in the cediranib-treated cohort. This does not preclude the possibility that treatment-induced hypoxia could have occurred earlier in the treatment time course counteracting a potential benefit of cediranib, which has been shown to modestly improve survival in an immunocompetent mouse glioma model. The discrepancy of these findings may be related to the time course in which we have evaluated hypoxia specifically during treatment failure as opposed to others that have evaluated hypoxia during early treatment initiation [37]. Alternatively, these tumors may already largely be hypoxic and subtle changes induced by antiangiogenic therapy [38] may not be detected with our assays. Indeed, it has been previously postulated that antiangiogenic agents would induce a reactive resistance mediated by the HIF-1/VEGF pathway [39]. Our results would support this contention since STAT3 is a known regulator of HIF-1/VEGF expression [40]. Finally, the expression of STAT3 in the setting of failed antiangiogenic therapy may be secondary to the induction of VEGF-independent angiogenic pathways [41] such as platelet derived growth factor, a known inducer of STAT3 [12].

AZD1480 demonstrated a reduction in microvascular density, likely secondary to STAT3 inhibition in the tumor vascular endothelium. However, the STAT3 pathway has been shown to be a potent regulator of the hypoxic pathway [20] and our *in vivo* data suggests that AZD1480 may circumvent the induction of hypoxia - a known mediator of treatment resistance to multiple cancer therapies. Cediranib alone did not appear to influence tumor hypoxia during treatment failure at the times evaluated in this model system, and no difference in CA9 expression was detected in the combination group. Cumulatively, these data indicate that tumor-associated hypoxia may induce STAT3 during the initiation of antiangiogenic therapy but certainly other mechanisms are involved.

The combination of cediranib with AZD1480 in the treatment of intracerebral glioma resulted in suppression of tumor growth. In the immune incompetent nude model of NS11, the combination of bevacizumab and AZD1480 resulted in only a modest increase in median survival. This was not entirely surprising in this murine model, since other STAT3 inhibitors (JSI-124 and WP1066) have previously been shown to have their therapeutic efficacy diminished in immune incompetent model systems [42]. Ideally, we would have liked to determine if AZD1480 could salvage the cediranib-treated cohort but secondary to the rapid animal demise from the time point of neurological symptoms to death (usually less than 48 hours), only a single dose of AZD1480 could be potentially administered. It would have been desirable to test AZD1480 in combination with bevacizumab in the GL261 model but because bevacizumab exclusively targets human VEGF, this combination experiment could only be done in human xenograft models that are immunologically incompetent. Significant therapeutic effects with anti-STAT3 agents are exerted through the immune system; thus, the therapeutic effect of AZD1480 is diminished. In the case of the cediranib, which has species cross reactivity, the combinational approach, as presented, is possible. Nonetheless, we would anticipate that the combination of bevacizumab with AZD1480 would also likely be beneficial in the setting of recurrent GB patients.

Our human data validated the observation of p-STAT3 up regulation during treatment failure of antiangiogenic therapy; however, only a limited number of patients were analyzed. The feasibility of collecting tumor tissue at the time of bevacizumab failure is limited owing to the relative contraindication of surgery within 28 days of administration of bevacizumab and the rapid deterioration of these patients. This is further confounded by a paucity of data to support a therapeutic benefit of re-resection in the setting of recurrent/progressive GB. Because surgery in close proximity to a recent dose of bevacizumab is contraindicated, we can't exclude that during the ensuing interval that there are changes in the human glioma p-STAT3 level. However, the murine models do not have this limitation and regardless of the model system or antiangiogneic therapy tested, we consistently observed up regulation of p-STAT3 levels.

It is highly likely that other pathways and mechanisms, in addition to the STAT3 pathway, are also involved during treatment failure of antiangiogenic therapy. For example, activation of proangiogenic growth factors and increased perivascular invasion has been proposed to contribute to tumor escape in this setting [6]. Furthermore, it is unknown if the withdraw of VEGF signaling inhibitors would result in the down regulation of p-STAT3 expression in the glioma microenvironment. The temporal relationship of peripheral blood p-STAT3 expression, treatment course, and recurrence are currently being investigated in a prospective clinical trial in patients receiving bevacizumab.

## MATERIALS AND METHODS

### Drugs

Bevacizumab (trade name Avastin, Genentech/Roche; Vacaville, CA) is a humanized monoclonal antibody that binds to VEGF-A [43], prevents interaction with the VEGF receptor [44], and was stored at a concentration of 25 mg/ml, in sterile PBS at 4°C and was further diluted in PBS immediately prior to use. Cediranib, also known as AZD2171, was provided by AstraZeneca and is a potent inhibitor of VEGF receptor tyrosine kinases [45], was stored as a powder at 4°C and formulated daily immediately prior to use by diluting in sterile water containing 1% Tween-80. AZD1480 is a potent inhibitor of the Jak2/STAT3 pathway [46] that has previously demonstrated a therapeutic effect in murine models of glioma [47].

### Tumor Cell Lines And Murine Models

The NSC11 cell line was derived from a GB patient that did not receive treatment with bevacizumab and has been previously described [48]. NSC11 cells were cultured in DMEM-F12 (1:1) media with B27 (Invitrogen, Carlsbad,CA), bFGF (Sigma, St. Louis, MO) and EGF (sigma, St. Louis, MO) at 37^°^ C in a humidified atmosphere of 5% CO_2_ and 95% air. GL261 cells were obtained from American Type Culture Collection and grown in 10% FBS (GIBCO-BRL, Rockville, Md.). All cell lines were grown in antibiotic-free medium and were free of *Mycoplasma* contamination.

For the *in vivo* experiments, we used 4- to 6-week-old female nude mice strictly inbred at The University of Texas M.D. Anderson Cancer Center (M.D. Anderson) and maintained in the M.D. Anderson Isolation Facility in accordance with Laboratory Animal Resources Commission standards and treated according to an approved protocol, 10-07-12132. To induce intracerebral tumors in mice, NSC11 or GL261 cells were collected in logarithmic growth phase, washed twice with PBS, mixed with an equal volume of 10% methyl cellulose in Improved MEM Zinc Option medium and loaded into a 250-μl syringe (Hamilton, Reno, NV) with an attached 25-gauge needle. The needle was positioned 2 mm to the right of bregma and 4 mm below the surface of the skull at the coronal suture using a stereotactic frame (Kopf Instruments, Tujunga, CA). The intracerebral tumorigenic dose for the NSC11 and GL261cells was 5 × 10^3^ and 5 × 10^4^, respectively, in a total volume of 5 μl. The mice are monitored daily and weighed at least three times per week during the treatment period. When the mice developed signs and symptoms of advanced tumors such as weight loss greater than 15%, neurological symptoms, or appeared distressed, they were compassionately euthanized with CO_2_ in accordance with animal welfare guidelines. The brain of each mouse was harvested, fixed in 4% formaldehyde, and embedded in paraffin. Tumor formation and the phenotype were determined by histologic analysis of H&E-stained sections. In order to determine tumor volume by external caliper, the greatest longitudinal diameter (length) and greatest transverse diameter (width) were determined. Tumor volumes were based on computer caliper measurements of digitized whole mount coronal CNS sections at the tumor injection site and were calculated by the modified ellipsoidal formula: Tumor volume = ½(length × width^2^) [49].

Treatment was started on day 5 after implantation of the glioma cells, and mice were treated via intraperitoneal (i.p.) injection with 10 mg/kg bevacizumab twice a week [50] or 6 mg/kg cediranib via oral gavage Monday through Friday. AZD1480 was formulated in 0.5% HPMC / 0.1% Tween 80 and administered by oral gavage once a day at 50 mg/kg Monday through Friday. These dosing schemas are typical for murine models that have very short median survivals compared to human patients. The control group was treated with PBS administered by i.p. injection or daily vehicle gavage. An IgG control group was also treated but the median survival was no different compared to the PBS group. Mice were treated continuously until death and there was no interruption between dosing and when the tissue was placed in formalin immediately after sacrifice. The time between last dose and tumor collection was never more than 2 days.

### Immunohistochemical analysis

Formalin-fixed, paraffin-embedded 4μm sections of the glioma were first deparaffinized in xylene and rehydrated in ethanol. Endogenous peroxidase was blocked with 0.3% hydrogen peroxide/methanol for 10 min at room temperature. Then, the ThermoScientific PTModule (Thermo Fisher Scientific, Fremont, CA) with citrate buffer (pH 6.0) was used for antigen retrieval. Immunohistochemical staining was performed using the primary antibody against p-STAT3 (Tyr705) antibody (1:50; Cell Signaling Technology, Danvers, MA), the macrophage-restricted cell surface glycoprotein F4/80 (1:50; Biolegend, San Diego, CA) and Nestin (1:300; Abcam, Cambridge, MA). The secondary antibodies used to label p-STAT3, F4/80 and Nestin were Alexa Fluor 488–conjugated anti-rabbit immunoglobulin G, Alexa Fluor 594-conjugated anti-rat immunoglobulin G and Alexa Fluor 594-conjugated anti-mouse immunoglobulin G (1:1000; Invitrogen, Carlsbad, CA), respectively. The expression p-STAT3, Nestin, and F4/80 were quantified by analyzing the tumors using high-power fields (max: x40 objective and x10 eyepiece) of each specimen. We counted the number of F4/80 or both F4/80 and p-STAT3 positively stained cells in the area of highest tumor cell density in nine non-overlapping high-power microscopic fields (at 200× magnification) from at least three different tumor-bearing brains from each group. The expression of Nestin was quantified by calculating the area of antibody staining per unit area of tumor using Photoshop CS4 software (Adobe).

Microvessel density (MVD) and area of hypoxia staining was determined by calculating the area of CD31 (1:50 dilution, BD Pharmingen, San Jose, CA) or Carbonic anhydrase 9 (Ca9) staining (Novus Biological, Littleton, CO), respectively, using the Image-Pro Plus system version 7.0 (Media Cybernetics) in × 20 fields of at least three tumor samples per group and three to fourth different sections per tumor sample.

### GB Tumor Staining for p-STAT3

Recurrent GB patients were identified retrospectively whom had received either conventional chemotherapy or treatment with bevacizumab that had failed treatment and who had undergone subsequent resection or biopsy at MDACC. Failure was defined as an increase in contrast enhancement and pathologically confirmed as recurrent tumor. The tumors were stained and quantified for p-STAT3 as previously described [13].

### Statistics

The distribution of p-STAT-3 positive cells was summarized by the mean, standard error and range. Due to the limited number of observations in murine models, the comparison of p-STAT-3 positive cells was conducted by Wilcoxon rank sum test if two samples are involved otherwise a Kruskal Wallis test was used if there were three-sample comparisons. Since there are multiple comparisons among the three different groups, type I error was adjusted based on Bonferroni correction (0.05/number of comparison=0.05/2=0.025). In another words, any p-value of 0.025 or smaller was considered statistical significant in the murine models [51]. Log rank tests were used to compare the difference between treatment and control. A Student's t test was used to compare the percent of p-STAT expressing cells between GB patients treated with bevacizumab and GB patients who had never been treated with any type of VEGF signaling inhibitors. All computations were carried out in SAS version 9.1 and S-Plus version 8.

## Supplementary Figures and Tables




