# Supplementary material for: Modulating Antiangiogenic Resistance by Inhibiting the Signal Transducer and Activator of Transcription 3 Pathway in Glioblastoma
Source: Oncotarget. 2012 Sep 19;3(9):1036–48. doi: 10.18632/oncotarget.663 (PMC3660053; doi:10.18632/oncotarget.663)
Supplement: Supplementary file 1 [file oncotarget-03-1036-s001.pdf]

Modulating Antiangiogenic Resistance by Inhibiting the Signal Transducer and Activator of Transcription 3 Pathway in Glioblastoma - *John de Groot*

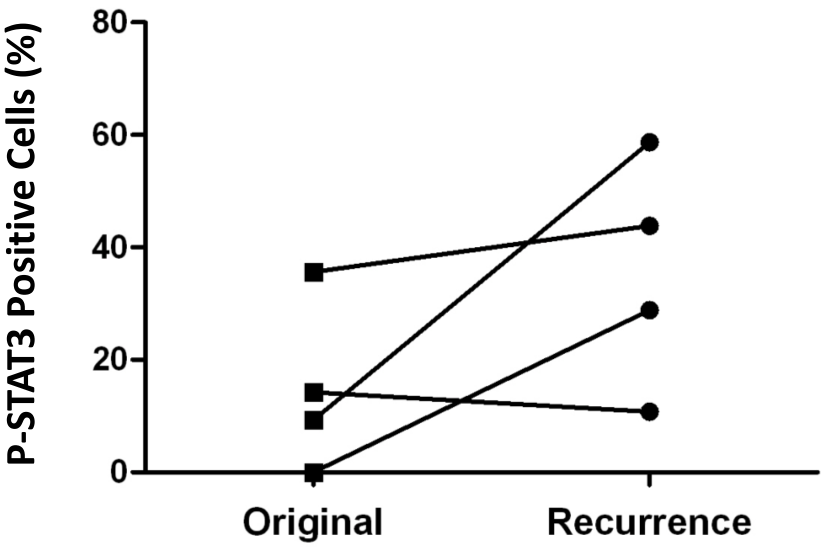

Supplementary Figure 1: Pairwise analysis of p-STAT3 expression from GB patients before and after receiving bevacizumab treatment.

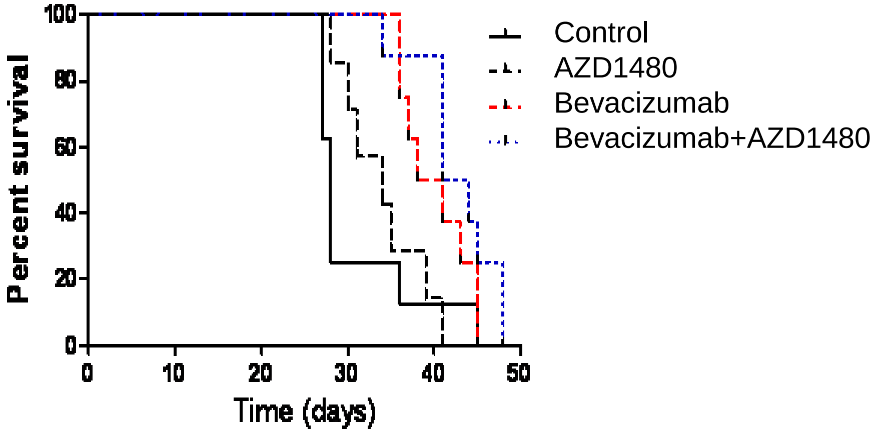

Supp. Fig. 2. Graph showing Kaplan-Meier estimate of survival showing improved survival in nude mice with NSC11 tumors treated with bevacizumab and AZD1480 compared with AZD1480 monotherapy, bevacizumab monotherapy or untreated controls.
