# Supplementary material for: Modulating Antiangiogenic Resistance by Inhibiting the Signal Transducer and Activator of Transcription 3 Pathway in Glioblastoma
Source: Oncotarget. 2012 Sep 19;3(9):1036–48. doi: 10.18632/oncotarget.663 (PMC3660053; doi:10.18632/oncotarget.663)
Supplement: Supplementary file 2 [file oncotarget-03-1036-s002.doc]

# Modulating Antiangiogenic Resistance by Inhibiting the Signal Transducer and Activator of Transcription 3 Pathway in Glioblastoma - John de Groot et al

**Supplementary Table -** Table of Prior Therapies

|  | | | | | |
| --- | --- | --- | --- | --- | --- |
| **Control** | **Original Surgery** | **RT+TMZ** | **Number of Adjuvant** | **Other Chemotherapy** | **STAT3 - Tissue Evaluation** |
|  | **(GTR/STR/Bx)** | **(Y/N)** | **TMZ Cycles** |  | **Time from Initial Diagnosis (months)** |
| 1 | Bx | Y | 17 | Accutane | 28 |
| 2 | STR | Y | 5 | None | 9 |
| 3 | STR | Y | 26 | None | 34 |
| 4 | STR | Y | 28 | None | 54 |
| 5 | STR | Y | 4 | None | 7 |
| 6 | STR | Y | 6 | None | 10 |
| 7 | STR | Y | 3 | None | 5 |
| 8 | GTR | Y | 12 | Accutane, Celebrex | 55 |
| 9 | STR | Y | 3 | None | 4 |
| 10 | GTR | Y | 0 | Accutane | 5 |
| 11 | GTR | Y | 12 | Accutane | 41 |
| 12 | GTR | Y | 10 | None | 12 |
| **Bevacizumab** | **Original Surgery** | **RT+TMZ** | **Number of Adjuvant** | **Other Chemotherapy** | **STAT3 - Tissue Evaluation** |
|  | **(GTR/STR/Bx)** | **(Y/N)** | **TMZ Cycles** |  | **Time from Initial Diagnosis (months)** |
| 1 | GTR | Y | 12 | Accutane, Irinotecan | 23 |
| 2 | STR | Y | 3 | None | 31 |
| 3 | STR | RT only | 4 | 6-thioguanine, lomustine, Xeloda, chloroquine, thalidomide, irinotecan | 26 |
| 4 | GTR | Y | 3 | 6-thioguanine, CCNU , Xeloda, Chloroquine, Hydroxyurea, Gleevec | 30 |
| 5 | STR | Y | 10 | None | 19 |
| 6 | STR | Y | 6 | Irinotecan | 41 |
| 7 | STR | Y | Unknown | RTA-744 | 27 |
| 8 | STR | Y | 12 | None | 13 |
